# Tracheotomy in the Advanced Stage of Croup

**Published:** 1845-01

**Authors:** 

**Affiliations:** Strasburg


FRENCH ACADEMY OF SCIENCES.

*Tracheotomy in the advanced stage of Croup.* By M. Scoutteten of Strasburg.—The patient was a child six weeks old. The symptoms at first were not very acute, but in the course of a day or two the danger of suffocation became very imminent. During the paroxysms, which became more and more frequent, attempts were made to produce vomiting, but with slight effect. Thus air was drawn by the mouth into the lungs of the patient, which caused the pulse to rally slightly, and animation to return. But this proving insufficient, a firm elastic sound was introduced into the larynx—any relief which it afforded, however, was before long neutralized by the cough and irritation excited by its presence. Only one resource was therefore left,—tracheotomy. It could scarcely aggravate the existing danger, and might, perhaps, if the substance of the lungs were healthy, avoid the impending suffocation. Accordingly, the operation was performed, and with the best possible results. On the tenth day after the operation, the tube was removed—the child's health became completely restored—and the tone of the voice appears to have suffered no alteration.

